# Supplementary material for: Propagation of viral genomes by replicating ammonia-oxidising archaea during soil nitrification
Source: ISME J. 2022 Nov 21;17(2):309–14. doi: 10.1038/s41396-022-01341-5 (PMC9859776; doi:10.1038/s41396-022-01341-5)
Supplement: Supplementary file 1 — Supplementary Information [file 41396_2022_1341_MOESM1_ESM.pdf]

Supplementary information

Propagation of viral genomes by replicating ammonia-oxidising archaea during soil nitrification

Sungeun Lee[1], Ella T. Sieradzki[1], Graeme W. Nicol[1]* and Christina Hazard[1]*

[1] Univ Lyon, CNRS, INSA Lyon, Université Claude Bernard Lyon 1, Ecole Centrale de Lyon, Ampère, UMR5005, 69134 Ecully, France

* Corresponding authors. Email: graeme.nicol@ec-lyon.fr
christina.hazard@ec-lyon.fr

Supplementary text

Materials and methods

Soil microcosms, DNA extraction and stable-isotope probing

Soil microcosms were established in 144 ml serum vial bottles with 10 g soil (dry weight equivalent). Microcosms were amended with urea solution (100 µg urea-N g^{-1} soil (dry weight)) at day 0 and 15 with the water content being 30% and 32% (w/w) after amendment, respectively. Headspace gas was amended with 5% (v/v) ^{12}C-CO_2 (Air Liquide) or ^{13}C-CO_2 (Sigma-Aldrich) (99% atom enriched) with microcosms opened every 3-4 days to maintain aerobic conditions before re-establishing CO_2 concentrations. Microcosms were destructively sampled at day 0, 15 and 30 in triplicate for each treatment and soil stored at -20°C. Ammonium, nitrite and nitrate concentrations were determined immediately after sampling using standard colorimetric assays [1]. Total genomic DNA was extracted from 0.5 g soil using a CTAB-buffer phenol:chloroform:isoamyl alcohol protocol [2] and subject to isopycnic centrifugation in CsCl gradients. Briefly, 8 ml polyallomer tubes were filled with CsCl dissolved in Tris EDTA buffer (buoyant density 1.71 g ml^{-1}) and 6 µg genomic DNA before sealing and centrifugation at 152,000 x *g* in an MLN80 rotor (Beckman-Coulter) at 20°C for 72 h. CsCl gradients were then fractionated into ~350 µl aliquots before determining buoyant density via refractive index and recovery of DNA by PEG precipitation [2]. The predicted GC mol% of DNA in specific fractions was determined using the calculation of Schildkraut *et al.* [3].

Quantitative PCR and metagenomic sequencing of fractionated DNA samples

Quantitative PCR (qPCR) was performed to determine the distribution of prokaryotic and nitrifier genomes through CsCl gradients using a Corbett Rotor-Gene 6000 real-

time PCR cycler. Prokaryotic 16S rRNA, bacterial and archaeal *amoA* genes were quantified across the entire buoyant density gradients using primer pairs P1(341f)/P2(534r) [4], amoA1F/amoA2R [5], and crenamoA23F/crenamoA616R [6]), respectively. Each 25 µl PCR contained 12.5 µl 2X QuantiFast SYBR Green Mix (Qiagen), 1 µM of each primer, 2 µl of standard DNA, recovered DNA from each fraction (1/10 dilution) or water (no template negative control). Thermal cycling programs consisted of an initial denaturation step of 15 min at 95°C, followed by 30 cycles of 15 s at 94°C, 30 s at 60°C, 30 s at 72°C for the 16S rRNA gene assay or 15 s at 94°C, 30 s at 55°C, 30 s at 72°C for bacterial and archaeal *amoA* gene assays followed by melt-curve analysis. All assays had an efficiency between 96 and 99% with an r^2 value ≥0.99. After assessing the distribution of prokaryotic and nitrifier genomes, DNA in fractions with a buoyant density between 1.676 to 1.699 g ml^{-1} (low buoyant density (LBD)) and 1.719 to 1.750 g ml^{-1} (high buoyant density (HBD)) were pooled for metagenome sequencing. LBD and HBD metagenomes were sequenced at IntegraGen (Paris, France) and the Joint Genome Institute (JGI, Berkeley, CA, USA), respectively, both using the NovaSeq sequencing platform (Illumina) with NovaSeq XP version 1 reagent kits and a S4 flowcell 150 bp paired reads.

Assembly, binning, and annotation

Individual reads of HBD metagenomes were quality-trimmed with the MetaWrap read_qc module [7]. Co-assembly of the 135 to 245 million quality-controlled reads per metagenome was performed using MEGAHIT version 1.2.9 [8]. Binning was performed using metaBAT 2 [9], MaxBin2 [10] and CONCOCT [11] implemented in MetaWRAP version 1.2.1 [7] and bin completion and contamination estimated using CheckM version 1.0.12 [12]. The relative abundance of sequences mapping to MAG

contigs in individual metagenomes was determined using default parameters in the MetaWRAP-Quant-bins module [7]. Taxonomic annotation of contigs was performed using Kaiju [13] with the NCBI nr database (2021-02-24). Taxonomy of MAGs was assigned using GTDB-Tk version 0.3.2 [14] with the Genome Taxonomy Database (GTDB, release date 2022-03-23).

Identification of putative viruses infecting nitrifiers

Viral contigs were predicted from contigs ≥10 kb using VirSorter [15], VirSorter 2.0 [16] and DeepVirFinder [17] (see Supplementary Figure 5 for comparison of predictions). CheckV [18] and manual curation (i.e. identification of viral hallmark genes such as capsid, portal, terminase and integrase proteins, enrichment in non-annotated genes) were performed to confirm a potential viral origin of each contig. The relative abundance of sequences mapping to virus contigs in individual metagenomes was determined using BBMap [19] with a threshold of ≥75% of contig length with ≥1x read coverage recruited at ≥90% average nucleotide identity [20]. The relative abundance of each viral contig in each HBD and LBD metagenome was calculated based on the length of sequence size and coverage and abundance was expressed as normalized copies per million reads (CPM). Gene prediction of each viral contig was performed using Prodigal version 2.6.3 with meta option [21], and homology search was performed against the NCBI nr database using Diamond blastp [22] and Interproscan5 [23].

To identify contigs from viruses infecting nitrifiers specifically, a database of virus genes present in proviruses of GTDB nitrifier reference genomes was constructed using VIBRANT [24], PhageBoost [25] and VirSorter [15] with 184 (87%) of 212 AOA genomes containing provirus regions. Capsid, terminase, portal and

integrase protein sequences were then used as search queries against each viral contig using Diamond blastp (e-value < 10^{-5}). Genes representing potential nitrifier-specific auxiliary metabolic genes were manually checked in annotation lists. A homologue-based approach was also used where an AOA host was predicted when 'best hit' shared homologues with those in genomes of *Thaumarchaeota* (NCBI taxonomy) were ≥3x more abundant compared to the second most dominant phylum [26]. Unlike our previous DNA-SIP analysis of methanotroph populations and associated viruses in these soils [27], spacer sequences in AOA MAG CRISPR arrays only matched predicted AOA viruses from other studies with a minimum of 2 mismatches between spacer and protospacer sequences and none from this study (data not shown).

Phylogenomic, phylogenetic and predicted protein analyses

Phylogenomic analysis of AOA MAGs and reference genomes from cultivated AOA and MAGs was performed using an alignment of single copy marker genes generated with GToTree [28] with a subsequent maximum likelihood phylogeny calculated using FastTree [29]. Single gene phylogenetic analyses were performed using alignments generated using MUSCLE [30] and manually refined. Maximum likelihood phylogenetic trees were constructed using PhyML [31] with automatic model selection. Affiliation of AOA MAGs to *amoA* gene-defined lineages was determined from phylogenetic analysis using the curated reference database of Alves *et al.* [32]. Where *amoA* genes were absent in incomplete *Nitrosotalea* MAGs, a broad-level affiliation was inferred from close phylogenomic relationships to complete genomes and their *amoA* lineage designation. Comparison of genome-wide similarity between AOA virus contigs and curated virus reference sequences was performed using ViPTree [33] and

the Virus-Host DB [34] to generate a protein tree based on normalised tBLASTx scores. Virus gene-sharing network analyses were performed using vConTACT v2.0 [35].

References

1. Hink L, Gubry-Rangin C, Nicol GW, Prosser JI. The consequences of niche and physiological differentiation of archaeal and bacterial ammonia oxidisers for nitrous oxide emissions. ISME J. 2018; 12: 1084–1093.
2. Nicol GW, Prosser JI. Strategies to determine diversity, growth, and activity of ammonia-oxidizing archaea in soil. Methods Enzymol. 2011;496:3–34.
3. Schildkraut CL, Marmur J, Doty P. Determination of the base composition of deoxyribonucleic acid from its buoyant density in CsCl. J Mol Biol. 1962;4:430–443.
4. Muyzer G, de Waal EC, Uitterlinden AG. Profiling of complex microbial populations by denaturing gradient gel electrophoresis analysis of polymerase chain reaction-amplified genes coding for 16S rRNA. Appl Environ Microbiol. 1993;59:695–700.
5. Rotthauwe JH, Witzel KP, Liesack W. The ammonia monooxygenase structural gene amoA as a functional marker: molecular fine-scale analysis of natural ammonia-oxidizing populations. Appl Environ Microbiol. 1997;63:4704–4712.
6. Tourna M, Freitag TE, Nicol GW, Prosser JI. Growth, activity and temperature responses of ammonia-oxidizing archaea and bacteria in soil microcosms. Environ Microbiol. 2008;10:1357–1364.
7. Uritskiy GV, DiRuggiero J, Taylor J. MetaWRAP-a flexible pipeline for genome-resolved metagenomic data analysis. Microbiome. 2018;6:1–13.

8. Li D, Liu C-M, Luo R, Sadakane K, Lam T-W. MEGAHIT: an ultra-fast single-node solution for large and complex metagenomics assembly via succinct de Bruijn graph. Bioinformatics. 2015;31:1674–1676.
9. Kang DD, Li F, Kirton E, Thomas A, Egan R, An H, et al. MetaBAT 2: an adaptive binning algorithm for robust and efficient genome reconstruction from metagenome assemblies. PeerJ. 2019;7:e7359.
10. Wu Y-W, Simmons BA, Singer SW. MaxBin 2.0: an automated binning algorithm to recover genomes from multiple metagenomic datasets. Bioinformatics. 2016;32:605–607.
11. Alneberg J, Bjarnason BS, de Bruijn I, Schirmer M, Quick J, Ijaz UZ, et al. Binning metagenomic contigs by coverage and composition. Nat Methods. 2014;11:1144–1146.
12. Parks DH, Imelfort M, Skennerton CT, Hugenholtz P, Tyson GW. CheckM: assessing the quality of microbial genomes recovered from isolates, single cells, and metagenomes. Genome Res. 2015;25:1043–1055.
13. Menzel P, Ng KL, Krogh A. Fast and sensitive taxonomic classification for metagenomics with Kaiju. Nat Commun. 2016;7: 11257.
14. Chaumeil P-A, Mussig AJ, Hugenholtz P, Parks DH. GTDB-Tk: a toolkit to classify genomes with the Genome Taxonomy Database. Bioinformatics. 2019;36: 1925–1927.
15. Roux S, Enault F, Hurwitz BL, Sullivan MB. VirSorter: mining viral signal from microbial genomic data. PeerJ. 2015;3:e985.
16. Guo J, Bolduc B, Zayed AA, Varsani A, Dominguez-Huerta G, Delmont TO, et al. VirSorter2: a multi-classifier, expert-guided approach to detect diverse DNA and RNA viruses. Microbiome. 2021;9:37.

17. Ren J, Song K, Deng C, Ahlgren NA, Fuhrman JA, Li Y, et al. Identifying viruses from metagenomic data using deep learning. Quant Biol. 2020;8:64–77.
18. Nayfach S, Camargo AP, Schulz F, Eloe-Fadrosh E, Roux S, Kyrpides NC. CheckV assesses the quality and completeness of metagenome-assembled viral genomes. Nat Biotechnol. 2021;39:578–585.
19. Bushnell B. BBMap: A fast, accurate, splice-aware aligner. 2014. Lawrence Berkeley National Lab. (LBNL), Berkeley, CA (United States).
20. Roux S, Adriaenssens EM, Dutilh BE, Koonin EV, Kropinski AM, Krupovic M, et al. Minimum Information about an Uncultivated Virus Genome (MIUViG). Nat Biotechnol. 2019;37:29–37.
21. Hyatt D, Chen G-L, Locascio PF, Land ML, Larimer FW, Hauser LJ. Prodigal: prokaryotic gene recognition and translation initiation site identification. BMC Bioinformatics. 2010;11:119.
22. Buchfink B, Xie C, Huson DH. Fast and sensitive protein alignment using DIAMOND. Nat Methods. 2015;12:59–60.
23. Jones P, Binns D, Chang H-Y, Fraser M, Li W, McAnulla C, et al. InterProScan 5: genome-scale protein function classification. Bioinformatics. 2014;30:1236–1240.
24. Kieft K, Zhou Z, Anantharaman K. VIBRANT: automated recovery, annotation and curation of microbial viruses, and evaluation of viral community function from genomic sequences. Microbiome. 2020;8:90.
25. Sirén K, Millard A, Petersen B, Gilbert MTP, Clokie MRJ, Sicheritz-Pontén T. Rapid discovery of novel prophages using biological feature engineering and machine learning. NAR Genom Bioinform. 2021;3:lqaa109.
26. Al-Shayeb B, Sachdeva R, Chen L-X, Ward F, Munk P, Devoto A, et al. Clades of huge phages from across Earth's ecosystems. Nature. 2020;578:425–431.

27. Lee S, Sieradzki ET, Nicolas AM, Walker RL, Firestone MK, Hazard C, et al. Methane-derived carbon flows into host–virus networks at different trophic levels in soil. Proc Natl Acad Sci U S A. 2021; 118: e2105124118.
28. Lee MD. GToTree: a user-friendly workflow for phylogenomics. Bioinformatics. 2019;35:4162–4164.
29. Price MN, Dehal PS, Arkin AP. FastTree 2 - approximately maximum-likelihood trees for large alignments. PLoS One. 2010;5:e9490.
30. Edgar RC. MUSCLE: multiple sequence alignment with high accuracy and high throughput. Nucleic Acids Res. 2004;32:1792–1797.
31. Guindon S, Dufayard J-F, Lefort V, Anisimova M, Hordijk W, Gascuel O. New algorithms and methods to estimate maximum-likelihood phylogenies: assessing the performance of PhyML 3.0. Syst Biol. 2010;59:307–321.
32. Alves RJE, Eloy Alves RJ, Minh BQ, Urich T, von Haeseler A, Schleper C. Unifying the global phylogeny and environmental distribution of ammonia-oxidising archaea based on amoA genes. Nat Commun. 2018;9:1517.
33. Nishimura Y, Yoshida T, Kuronishi M, Uehara H, Ogata H, Goto S. ViPTree: the viral proteomic tree server. Bioinformatics. 2017;33:2379–2380.
34. Mihara T, Nishimura Y, Shimizu Y, Nishiyama H, Yoshikawa G, Uehara H, et al. Linking virus genomes with host taxonomy. Viruses. 2016;8:66.
35. Bin Jang H, Bolduc B, Zablocki O, Kuhn JH, Roux S, Adriaenssens EM, et al. Taxonomic assignment of uncultivated prokaryotic virus genomes is enabled by gene-sharing networks. Nat Biotechnol. 2019;37:632–639.

Supplementary Table 1. Summary of metagenome sequencing of genomic DNA from low buoyant density (LBD) and high buoyant density (HBD) CsCl fractions derived from pH 4.5 and 7.5 soil. **A** Library accession numbers and read depth. **B** Contig assembly details.

A

Sample ID*	NCBI BioProject Accession	Post-QC number of reads
LBD-12C-pH45_1	PRJNA868779	170,519,430
LBD-12C-pH45_2	PRJNA868779	190,442,846
LBD-12C-pH45_3	PRJNA868779	183,142,278
LBD-12C-pH75_1	PRJNA868779	178,892,472
LBD-12C-pH75_2	PRJNA868779	180,678,072
LBD-12C-pH75_3	PRJNA868779	165,641,626
HBD-12C-pH45_1	PRJNA621418	137,927,826
HBD-12C-pH45_2	PRJNA621419	165,499,900
HBD-12C-pH45_3	PRJNA621420	161,786,148
HBD-13C-pH45_1	PRJNA621421	157,372,294
HBD-13C-pH45_2	PRJNA621422	140,157,446
HBD-13C-pH45_3	PRJNA621423	153,263,184
HBD-12C-pH75_1	PRJNA621424	172,033,988
HBD-12C-pH75_2	PRJNA621425	184,944,656
HBD-12C-pH75_3	PRJNA621426	173,060,618
HBD-13C-pH75_1	PRJNA621427	135,351,318
HBD-13C-pH75_2	PRJNA621428	245,188,756
HBD-13C-pH75_3	PRJNA621429	156,686,154

*name describes HBD or LBD CsCl fractions, ^{12}C or ^{13}C incubation, soil pH and replicate number.

B

Sample ID	Assembled contigs	Mean contig length (bp)	Max. contig length (bp)
LBD-co-assembled contig	26,611,526	614.2	809,772
≥5kb-LBD-co-assembled contig	117,750	9,437.5	
HBD-co-assembled contig	35,109,680	566.3	812,893
≥5kb-HBD-co-assembled contig	76,311	8,810.5	

Supplementary Table 2. Summary of 123 medium- and high-quality metagenome assembled genomes from low buoyant density DNA ranked by phylum (GTDB taxonomy).

MAG ID	Completeness (%)	Contamination (%)	GC mol%	N50 (bp)	Size (bp)	Phylum	Class	Order	Family	Genus
75	83.7	0.2	66.9	24,140	2,864,512	Acidobacteriota	Acidobacteriae	Acidobacteriales		
56	82.5	0.0	60.3	15,391	3,027,569	Acidobacteriota	Acidobacteriae	Acidobacteriales	Acidobacteriaceae	PALSA-350
101	80.7	5.1	54.0	21,626	3,847,244	Acidobacteriota	Acidobacteriae	Acidobacteriales	Koribacteraceae	Gp1-AA145
126	77.1	3.0	67.5	17,321	3,322,481	Acidobacteriota	Vicinamibacteria	Vicinamibacterales	UBA2999	
72	73.9	0.9	58.4	14,749	2,220,118	Acidobacteriota	Acidobacteriae	UBA7541	UBA7541	
124	67.3	3.4	63.9	11,528	2,512,459	Acidobacteriota	Acidobacteriae	Acidobacteriales		
33	66.0	5.5	59.3	15,405	2,887,037	Acidobacteriota	Acidobacteriae	UBA7541	UBA7541	Palsa-295
22	53.3	0.9	56.3	9,893	2,452,695	Acidobacteriota	Acidobacteriae	UBA7541	UBA7541	
68	50.8	3.4	61.9	9,900	2,500,689	Acidobacteriota	Acidobacteriae	Acidobacteriales	Acidobacteriaceae	KBS-83
87	50.4	2.0	52.4	8,779	1,812,072	Acidobacteriota	Blastocatellia	Pyrinomonadales	Pyrinomonadaceae	OLB17
111	97.3	1.9	66.6	36,117	2,871,784	Actinobacteriota	Thermoleophilia	Solirubrobacterales	Solirubrobacteraceae	
44	97.0	2.6	66.0	142,875	3,274,599	Actinobacteriota	Thermoleophilia	Solirubrobacterales	Solirubrobacteraceae	
25	97.0	3.1	65.7	71,608	2,181,555	Actinobacteriota	Thermoleophilia	Solirubrobacterales	70-9	
46	82.0	0.1	63.9	17,436	1,359,101	Actinobacteriota	Acidimicrobiia	Acidimicrobiales	RAAP-2	RAAP-2
6	79.2	2.2	68.5	54,707	2,098,665	Actinobacteriota	Thermoleophilia	Solirubrobacterales	70-9	
57	70.8	1.2	69.6	12,884	2,594,437	Actinobacteriota	Actinobacteria	Nanopelagicales		
48	63.0	3.0	70.1	11,121	2,066,628	Actinobacteriota	Acidimicrobiia	Acidimicrobiales	RAAP-2	Bog-756
93	60.3	0.9	62.8	10,343	1,801,421	Actinobacteriota	Acidimicrobiia	Microtrichales	Ilumatobacteraceae	UBA668
79	67.5	0.9	61.7	11,258	2,006,440	Armatimonadota	Fimbriimonadia	Fimbriimonadales	Fimbriimonadaceae	
38	96.5	0.7	44.9	52,178	3,501,364	Bacteroidota	Bacteroidia	Chitinophagales	Chitinophagaceae	JJ008
100	94.8	1.5	39.6	298,057	4,276,415	Bacteroidota	Bacteroidia	AKYH767		
53	92.0	1.7	44.7	26,881	3,591,767	Bacteroidota	Bacteroidia	Chitinophagales	Chitinophagaceae	UBA8621
62	86.9	7.5	38.5	28,454	3,315,570	Bacteroidota	Bacteroidia	AKYH767-A	OLB10	
76	84.0	0.7	49.6	26,115	3,041,112	Bacteroidota	Bacteroidia	AKYH767	2-12-FULL-35-15	
97	78.6	2.1	39.6	15,282	3,408,942	Bacteroidota	Bacteroidia	AKYH767		
81	77.5	0.2	54.5	15,405	2,291,910	Bacteroidota	Bacteroidia	Chitinophagales	Chitinophagaceae	
106	76.6	3.4	44.4	12,939	2,749,566	Bacteroidota	Bacteroidia	AKYH767	b-17BO	PALSA-968
32	75.2	3.8	40.6	13,274	4,299,582	Bacteroidota	Bacteroidia	AKYH767-A	OLB10	
47	73.6	4.2	34.8	11,826	2,228,512	Bacteroidota	Ignavibacteria	Ignavibacteriales	Ignavibacteriaceae	BMS3ABIN03
116	73.2	1.9	51.5	13,585	2,332,193	Bacteroidota	Kapabacteria	Palsa-1295	Palsa-1295	
13	71.5	0.0	42.1	13,719	2,460,739	Bacteroidota	Bacteroidia	NS11-12g	UBA955	
30	67.2	0.5	37.7	10,543	1,634,027	Bacteroidota	Bacteroidia	Sphingobacteriales	Sphingobacteriaceae	
26	66.5	1.5	42.0	11,489	3,546,402	Bacteroidota	Bacteroidia	Chitinophagales	Chitinophagaceae	
86	65.7	3.4	34.7	9,330	2,102,202	Bacteroidota	Bacteroidia	Flavobacteriales	Flavobacteriaceae	Flavobacterium
89	65.4	0.0	39.1	18,038	2,305,465	Bacteroidota	Ignavibacteria	SJA-28	OLB5	
40	64.5	7.3	39.0	8,876	2,679,712	Bacteroidota	Bacteroidia	Chitinophagales	Chitinophagaceae	Taibaiella_B
118	52.0	3.2	38.0	9,613	3,001,274	Bacteroidota	Bacteroidia	Chitinophagales	Chitinophagaceae	Palsa-955
41	51.3	4.4	39.4	8,197	2,857,311	Bacteroidota	Bacteroidia	Chitinophagales	BACL12	UBA7236
83	50.8	1.0	41.9	11,602	2,718,158	Bacteroidota	Bacteroidia	Chitinophagales	Chitinophagaceae	
49	52.6	1.8	42.5	10,353	1,916,736	Bdellovibrionota	Bdellovibrionia	Bdellovibrionales	Bdellovibrionaceae	Bdellovibrio
58	91.4	0.0	61.2	14,550	3,678,188	Chloroflexota	UBA5177	UBA5177	UBA5177	
69	75.9	2.2	62.3	12,810	2,934,925	Chloroflexota	UBA4733	UBA4733	UBA4733	
115	75.1	2.8	70.3	18,220	2,481,858	Chloroflexota	Ellin6529	CSP1-4	CSP1-4	Palsa-1033
16	64.2	1.4	55.1	23,141	1,640,525	Chloroflexota	Anaerolineae	Anaerolineales	UBA11579	
121	63.5	8.2	48.7	8,645	3,693,765	Chloroflexota	Anaerolineae	Anaerolineales	envOPS12	OLB14
14	62.9	1.0	68.7	11,300	3,380,230	Chloroflexota	Ktedonobacteria	Ktedonobacterales		

36	59.5	2.8	72.0	10,127	1,826,549	Chloroflexota	Ellin6529	CSP1-4	CSP1-4	CSP1-4
17	57.0	0.9	64.1	9,099	3,136,804	Chloroflexota	UBA5177			
24	56.3	0.9	63.3	7,671	2,328,854	Chloroflexota	UBA5177			
43	92.3	0.9	43.4	23,016	2,895,603	Cyanobacteria	Vampirovibrionia	Vampirovibrionales		
104	52.6	0.0	46.6	9,501	1,686,795	Cyanobacteria	Vampirovibrionia	Vampirovibrionales		
84	62.1	0.0	35.2	9,392	965,279	Dependentiae	Babeliae	Babeliales		
11	60.2	0.9	69.4	14,745	1,971,573	Dormibacterota	Dormibacteria	UBA8260	UBA8260	
23	54.3	9.5	68.9	8,592	1,357,015	Dormibacterota	Dormibacteria	UBA8260	UBA8260	Palsa-851
119	87.2	0.0	38.0	15,691	2,341,565	Eremiobacterota	Eremiobacteria	Eremiobacterales	Eremiobacteraceae	
103	77.4	1.5	68.2	12,265	2,115,044	Eremiobacterota	Eremiobacteria	UBP12	UBA5184	
37	51.1	7.4	60.2	7,077	1,974,572	Eremiobacterota	Eremiobacteria	UBP12	UBA5184	PALSA-1484
82	95.1	0.3	34.9	35,039	2,302,984	Firmicutes	Clostridia	Lachnospirales	Lachnospiraceae	Herbinix
70	84.4	2.6	41.4	17,334	3,226,646	Firmicutes	Bacilli	Bacillales	Bacillaceae	
10	78.7	0.8	45.6	21,297	2,470,344	Firmicutes	Bacilli	Bacillales	Bacillaceae	
123	59.2	1.0	36.4	18,393	979,880	Firmicutes	Bacilli	Bacillales	Planococcaceae	Ureibacillus
99	54.5	9.9	36.8	6,988	796,782	Firmicutes	Bacilli	Bacillales	Bacillaceae_G	Bacillus
3	51.9	5.3	32.6	12,001	1,498,041	Firmicutes	Clostridia	Acetivibrionales	Acetivibrionaceae	Herbivorax
80	97.4	0.7	41.7	35,221	2,034,588	Firmicutes_F	Halanaerobiia	Halanaerobiales	DTU029	DTU029
35	99.2	1.0	42.0	79,014	2,517,665	Firmicutes_I	Bacilli	Thermoactinomycetales	Thermoactinomycetaceae	
120	96.5	0.3	48.2	35,048	2,270,355	Firmicutes_I	Bacilli	Thermoactinomycetales	Thermoactinomycetaceae	CDF
67	69.4	0.3	41.6	9,016	1,357,832	Firmicutes_I	Bacilli	Thermoactinomycetales	Thermoactinomycetaceae	
122	71.8	2.7	65.3	16,326	2,944,945	Gemmatimonadota	Gemmatimonadetes	Gemmatimonadales	Gemmatimonadaceae	FEN-1250
34	71.6	3.4	66.1	29,597	3,506,635	Gemmatimonadota	Gemmatimonadetes	Gemmatimonadales	Gemmatimonadaceae	
39	69.1	1.2	67.2	17,110	3,202,677	Gemmatimonadota	Gemmatimonadetes	Gemmatimonadales	Gemmatimonadaceae	
91	52.7	0.0	62.8	9,236	2,678,084	Gemmatimonadota	Gemmatimonadetes	Gemmatimonadales	Gemmatimonadaceae	AG2
117	58.9	0.0	68.6	7,421	2,581,217	Myxococcota	Polyangia	Palsa-1104	Palsa-1104	PALSA-1104
31	78.3	0.0	35.0	63,246	1,110,445	Patescibacteria	Dojkabacteria			
110	72.5	0.0	45.6	538,338	833,363	Patescibacteria	Saccharimonadia	Saccharimonadales	2-12-FULL-41-12	
102	70.0	0.0	35.8	24,450	1,170,104	Patescibacteria	Microgenomatia	Levybacterales	UBA12049	
60	69.9	1.9	63.1	809,772	809,772	Patescibacteria	Saccharimonadia			
51	65.0	0.8	58.3	36,742	983,527	Patescibacteria	Paceibacteria	UBA6257	2-01-FULL-56-20	
50	64.6	0.0	39.7	277,019	821,951	Patescibacteria	Microgenomatia	UBA1406	HO2-37-13b	
66	64.5	0.0	41.9	35,340	846,808	Patescibacteria	Microgenomatia	UBA1406	GWC2-37-13	2-01-FULL-40-42
42	64.4	0.0	43.7	72,802	632,826	Patescibacteria	Saccharimonadia	Saccharimonadales	UBA10212	
7	64.2	0.0	53.8	40,147	643,112	Patescibacteria	Saccharimonadia	Saccharimonadales	UBA10212	
29	60.9	1.0	43.9	26,687	688,051	Patescibacteria	Doudnabacteria	UBA920	O2-02-FULL-48-8	
45	59.9	0.0	39.8	12,821	852,036	Patescibacteria	Microgenomatia	Levybacterales	UBA12049	PRDT01
28	59.4	0.0	49.9	16,270	829,123	Patescibacteria	Saccharimonadia	Saccharimonadales	UBA4665	UBA6224
74	54.6	0.0	35.5	26,797	536,454	Patescibacteria	Microgenomatia	Levybacterales	UBA12049	
114	53.4	6.5	43.9	9,739	841,384	Patescibacteria	Saccharimonadia	Saccharimonadales	2-12-FULL-41-12	
27	52.7	0.4	46.8	40,409	442,116	Patescibacteria	Paceibacteria	UBA9983	Zambryskibacteraceae	UBA5004
5	51.9	0.0	37.5	13,915	425,834	Patescibacteria	Paceibacteria	UBA9983	Zambryskibacteraceae	C7867-006
54	50.4	0.0	38.2	21,102	760,548	Patescibacteria	Microgenomatia	Woykebacterales		
8	66.5	0.0	58.4	10,752	3,564,280	Planctomycetota	Phycisphaerae	UBA1161	UBA1161	
105	65.4	5.8	59.4	11,513	4,165,716	Planctomycetota	Phycisphaerae	UBA1161	UBA1161	
73	95.1	4.3	65.6	137,474	4,398,083	Proteobacteria	Gammaproteobacteria	Steroidobacterales	Steroidobacteraceae	
19	89.5	2.6	70.1	31,514	3,812,969	Proteobacteria	Alphaproteobacteria	Caulobacterales	Caulobacteraceae	BOG-938
125	88.0	3.1	62.4	28,686	2,191,964	Proteobacteria	Alphaproteobacteria	Sphingomonadales	Sphingomonadaceae	Sphingomonas
108	87.5	6.2	57.0	22,826	1,972,878	Proteobacteria	Alphaproteobacteria	Micavibrionales	Micavibrionaceae	UM-FILTER-47-13
92	82.8	5.0	65.9	19,149	2,660,651	Proteobacteria	Gammaproteobacteria	Steroidobacterales	Steroidobacteraceae	13-2-20CM-66-19
64	81.0	1.7	61.6	22,864	2,553,403	Proteobacteria	Gammaproteobacteria	Xanthomonadales	Rhodanobacteraceae	Rudaea
71	79.5	0.9	61.2	22,385	2,981,098	Proteobacteria	Alphaproteobacteria	UBA1301	UBA1301	UBA6038

98	79.3	2.1	70.5	22,416	2,489,191	Proteobacteria	Gammaproteobacteria	Steroidobacterales	Steroidobacteraceae	13-2-20CM-66-19
94	78.7	0.9	59.3	14,277	2,998,862	Proteobacteria	Alphaproteobacteria	Rhizobiales	Xanthobacteraceae	Pseudolabrys
95	77.7	0.0	35.2	13,606	3,485,685	Proteobacteria	Gammaproteobacteria	UBA5158	UBA5158	
65	77.3	1.1	66.2	16,642	5,023,705	Proteobacteria	Alphaproteobacteria	Acetobacterales	Acetobacteraceae	Palsa-883
2	73.3	1.4	67.0	20,592	2,309,631	Proteobacteria	Alphaproteobacteria	Sphingomonadales	Sphingomonadaceae	Porphyrobacter
109	61.9	5.3	63.3	13,874	3,496,047	Proteobacteria	Alphaproteobacteria	Rhizobiales	Xanthobacteraceae	BOG-931
78	56.7	1.3	64.9	35,205	1,728,632	Proteobacteria	Alphaproteobacteria	Elsterales	URHD0088	
55	56.4	0.8	61.4	11,298	1,368,874	Proteobacteria	Alphaproteobacteria	Rhizobiales	Methyloligellaceae	Methyloceanibacter
90	55.5	2.1	67.2	11,614	1,755,960	Proteobacteria	Gammaproteobacteria	Burkholderiales	Palsa-1005	PALSA-1003
88	55.2	3.4	65.1	9,131	1,518,294	Proteobacteria	Alphaproteobacteria	Rhizobiales	Xanthobacteraceae	Palsa-892
18	52.5	5.7	59.0	7,355	2,371,465	Proteobacteria	Alphaproteobacteria	Rhizobiales	Methyloligellaceae	Methyloceanibacter
20	51.7	0.0	61.0	9,057	2,689,297	Proteobacteria	Alphaproteobacteria	UBA1301	UBA1301	
15	51.2	1.7	59.9	9,856	2,680,085	Proteobacteria	Alphaproteobacteria	Rhizobiales	Xanthobacteraceae	Nitrobacter
21	50.3	1.1	64.3	8,593	1,496,296	Proteobacteria	Gammaproteobacteria	Burkholderiales	Burkholderiaceae	C04
96	91.3	1.9	31.0	10,635	4,039,103	Thermoproteota	Nitrososphaeria	Nitrososphaerales	Nitrososphaeraceae	TH1177
52	88.8	1.9	49.7	15,865	1,740,480	Thermoproteota	Nitrososphaeria	Nitrososphaerales	Nitrososphaeraceae	Nitrososphaera
61	87.9	1.9	37.8	14,936	1,491,779	Thermoproteota	Nitrososphaeria	Nitrososphaerales	Nitrosopumilaceae	Nitrosotalea
107	85.9	2.9	37.8	36,213	1,658,330	Thermoproteota	Nitrososphaeria	Nitrososphaerales	Nitrosopumilaceae	Nitrosotalea
63	78.6	1.0	37.4	10,792	2,066,632	Thermoproteota	Nitrososphaeria	Nitrososphaerales	Nitrososphaeraceae	
12	73.3	3.2	50.2	11,601	1,019,284	Thermoproteota	Nitrososphaeria	Nitrososphaerales	Nitrososphaeraceae	Nitrososphaera
77	65.2	6.5	36.8	16,902	1,553,150	Thermoproteota	Nitrososphaeria	Nitrososphaerales	Nitrosopumilaceae	Nitrosotalea
112	62.9	1.0	37.9	9,638	1,212,031	Thermoproteota	Nitrososphaeria	Nitrososphaerales	Nitrososphaeraceae	TA-21
9	61.7	1.0	37.3	13,015	970,273	Thermoproteota	Nitrososphaeria	Nitrososphaerales	Nitrosopumilaceae	Nitrosotalea
85	98.6	8.6	57.1	36,458	4,399,804	Verrucomicrobiota	Verrucomicrobiae	Pedosphaerales	Pedosphaeraceae	UBA11358
59	94.6	1.6	37.8	26,369	2,935,834	Verrucomicrobiota	Chlamydiia	Parachlamydiales	Parachlamydiaceae	

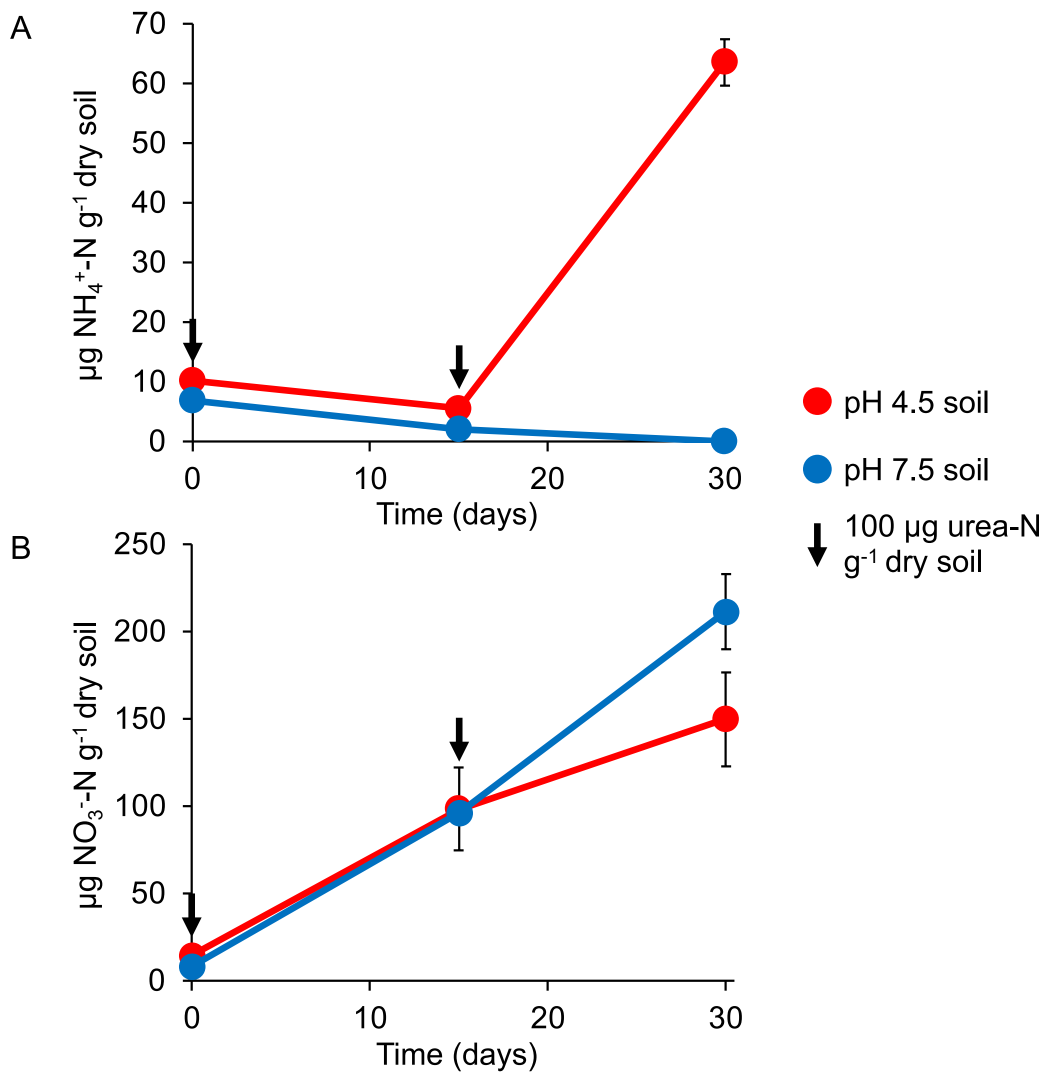

Supplementary Figure 1. Nitrification kinetics in soil microcosms. **A** Ammonium and **B** Nitrite+nitrate concentrations in pH 4.5 and 7.5 soil microcosms after addition of 2 x 100 μg urea-N g^{-1} soil. Ammonia concentrations were determined prior to the addition of urea on day 0 and 15. Points and error bars represent the mean and standard error value from three destructively sampled microcosms, with some error bars smaller than the symbol. Microcosms were incubated with $^{12}C\text{-}CO_2$ for determining nitrification kinetics with no significant differences observed with N concentrations in ^{13}C microcosms at day 30 (data not shown).

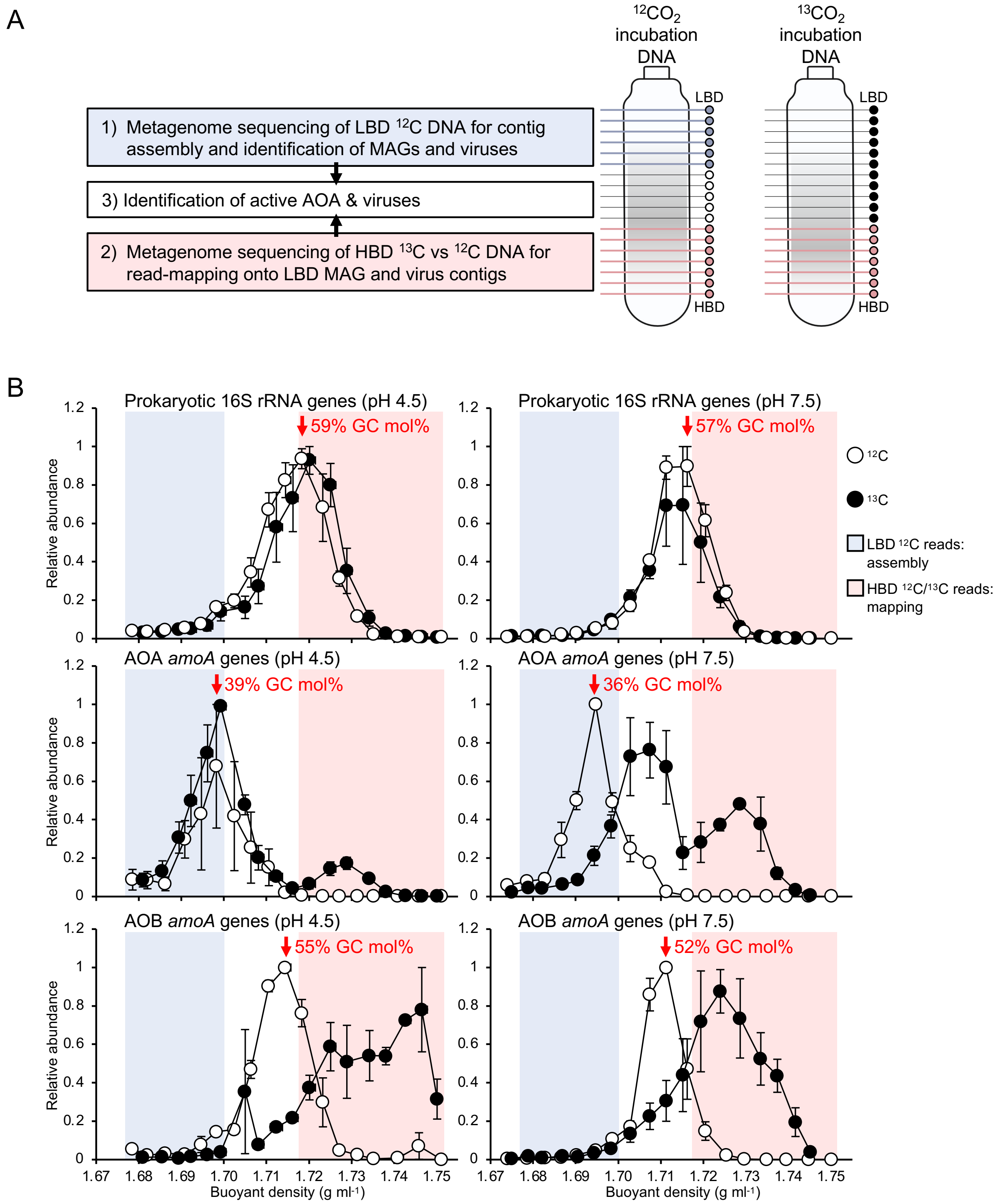

Supplementary Figure 2. Isopycnic centrifugation and metagenomic sequencing of DNA in targeted fractions for hybrid analysis of ^{12}C- and ^{13}C-enriched DNA. **A** Schematic of GC mol% fractionation in CsCl and selection of samples for metagenomic sequencing and analysis. **B** Distribution of total prokaryotic, AOA and AOB genomes in CsCl gradients determined from the relative abundance of 16S rRNA genes (prokaryotes) and *amoA* genes (AOA and AOB). Vertical error bars are the standard error of the mean relative abundance and horizontal bars (mostly smaller than the symbol size) the standard error of the mean buoyant density of individual fractions from three independent CsCl gradients, each representing an individual microcosm. Fractions highlighted in blue or pink areas were pooled for each replicate microcosm for sequencing. The GC mol% of genomic DNA is given (red text and arrow) for the ^{12}C fraction containing the highest quantity of genomic DNA for each target group.

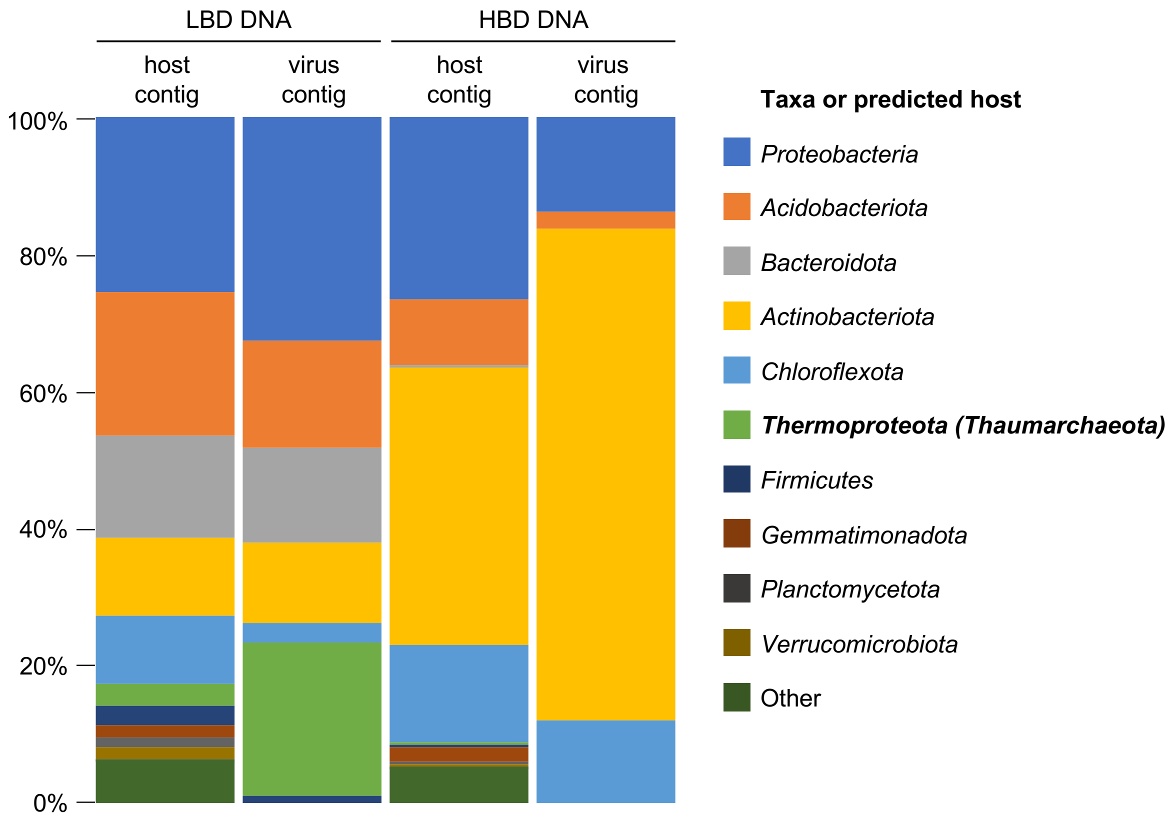

Supplementary Figure 3. Taxonomic affiliation (GTDB) of contigs from prokaryote genomes (host contigs) or predicted hosts of viruses (virus contigs) in low buoyant density (LBD) and high buoyant density (HBD) metagenomic libraries. Unaffiliated contigs are not included. AOA of the class *Nitrososphaeria* are placed within the *Thermoproteota* (GTDB), *Nitrososphaerota* (ICNP) or *Thaumarchaeota* (NCBI) phylum. Data are the mean values from both pH 4.5 and 7.5 libraries.

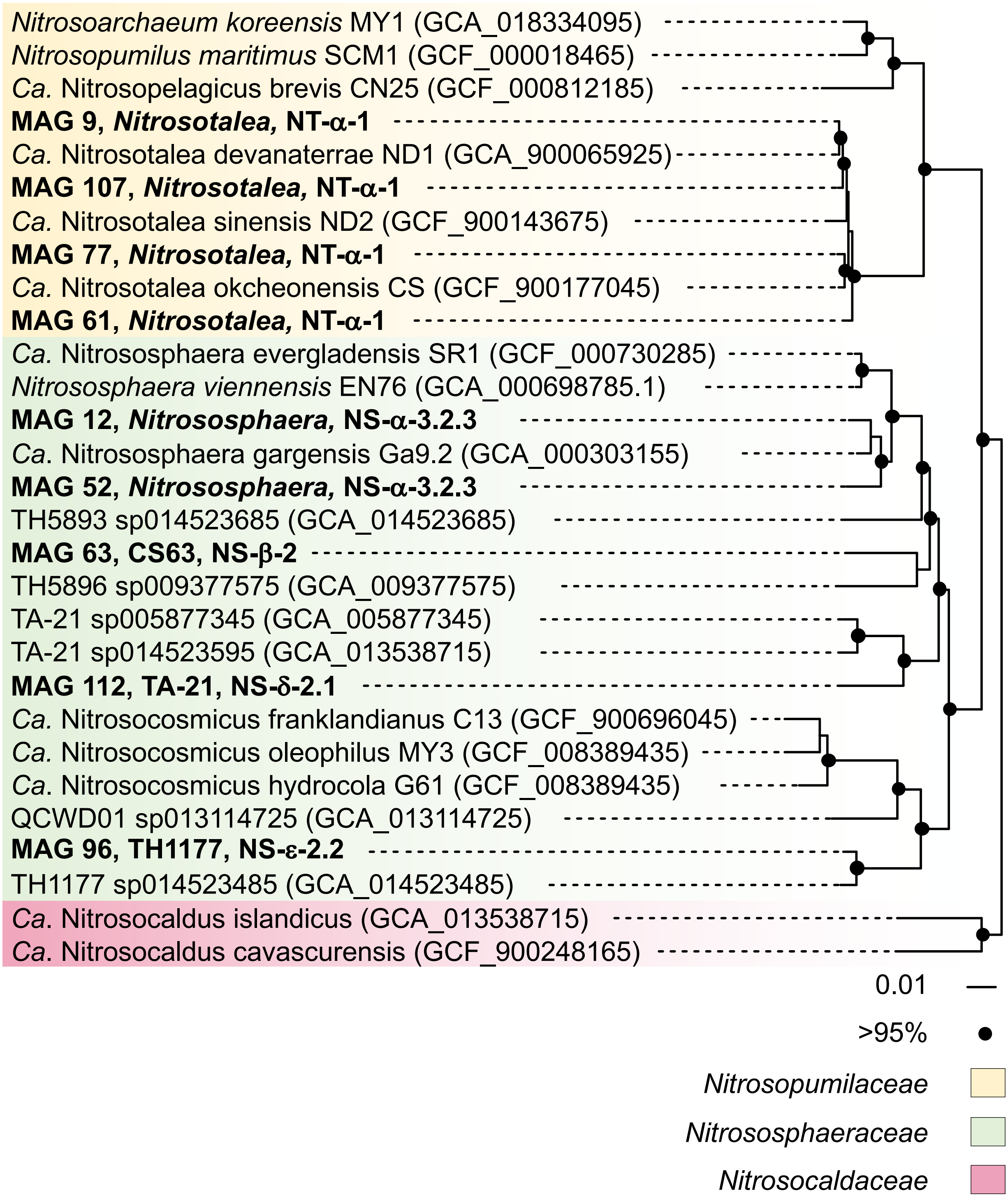

Supplementary Figure 4. Maximum likelihood phylogenomic analysis of nine AOA MAGs within the class *Nitrososphaeria*. MAGs were compared with cultivated AOA genomes or reference MAGs using 14,524 aligned positions from 76 single copy genes and rooted with *Nitrosocaldus* representatives. NCBI accession numbers are given in parenthesis. Circles at nodes represent >95% percentage bootstrap support from 1000 replicates and the scale bar denotes an estimated 0.01 changes per position.

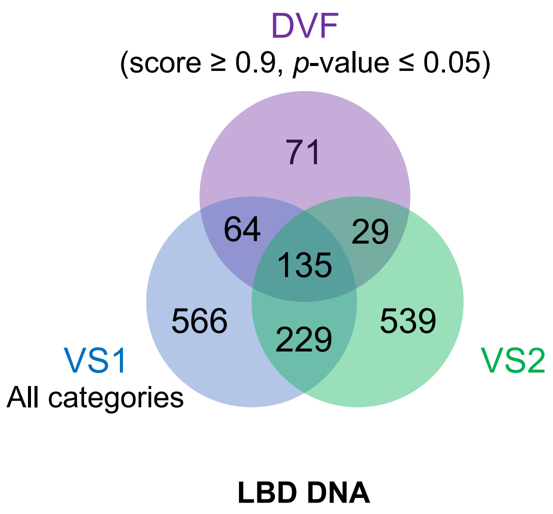

Supplementary Figure 5. Venn diagrams comparing the number of predicted virus contigs using VirSorter1, VirSorter2 and DeepVirFinder tools from low buoyant density (LBD) and high buoyant density (HBD) DNA metagenomic libraries from both pH 4.5 and 7.5 soils.
